# Maternal Adiposity and Inflammation: Risk Factors for Iron Deficiency in Pregnancy

**DOI:** 10.1016/j.tjnut.2025.08.022

**Published:** 2025-08-29

**Authors:** Sabrina P Demirdjian, Maria S Mulhern, Maeve A Kerr, Mark Ledwidge, Raghad M Alhomaid, Paul D Thompson, Mary T McCann

**Affiliations:** 1 Nutrition Innovation Centre for Food and Health, School of Biomedical Sciences, Ulster University, Coleraine, Northern Ireland; 2 School of Medicine, University College Dublin, Ireland; 3 Department of Food Science and Human Nutrition, College of Agriculture and Food, Qassim University, Buraydah, Saudi Arabia

**Keywords:** anemia, body fat, iron markers, visceral fat, interleukins, iron depletion, hypoferremia, iron status, prenatal care, gravidity

## Abstract

**Background:**

Obesity and iron deficiency (ID) are global health concerns in pregnancy, with serious consequences for mother and offspring. The inflammatory state associated with obesity and its potential contribution to ID/anemia is unclear.

**Objectives:**

This study aims to investigate the associations among maternal adiposity, the mediating role of inflammation, and iron status. We also aim to examine how adiposity affects the predictive accuracy of early pregnancy iron markers for late pregnancy ID risk.

**Methods:**

This secondary analysis of a double-blind randomized controlled trial included singleton pregnancies supplemented with a multivitamin containing 17 mg/d of iron. Body mass index (BMI), body composition [12 gestational weeks (GW)], iron markers (12, 28, 36 GW), and hemoglobin/hematological indices (12, 28 GW, postpartum) were assessed. Proinflammatory cytokines were used to calculate an inflammation score and categorized as high/low inflammation.

**Results:**

A total of 125 pregnant women were included: 43 normal weight, 44 overweight, and 38 with obesity. At 36 GW, ID was present in 50% of women with obesity, 40.9% of those overweight, and 30.2% with normal BMI. High BMI and fat mass index (FMI) at 12 GW predicted lower ferritin at 36 GW (BMI *β* = –0.253, *P* = 0.020; FMI *β* = –0.265, *P* = 0.010), and all adiposity measures predicted higher soluble transferrin receptor (sTfR). Transferrin saturation was lower in women with obesity at 12 and 28 GW (12 GW 23.9%, 24.5%, 30.3%, *P* = 0.016; 28 GW 13.2%, 17.7%, 17.2% *P* < 0.001, obesity, overweight, and normal weight, respectively). At 36 GW, pregnant women with obesity and higher inflammation score had lower ferritin than normal weight women (15.0 compared with 20.3 μg/L, *P* = 0.041). sTfR at 12 GW was the best predictor of ID at 36 GW [area under curve (AUC) = 0.738, *P* < 0.001], especially in overweight/obesity (AUC = 0.744, *P* < 0.001).

**Conclusions:**

High adiposity, mediated by inflammation, increases the risk of ID in the late third trimester. sTfR in early pregnancy emerges as an effective marker for predicting ID in late pregnancy.

## Introduction

Anemia is a global health problem affecting 1.92 billion people, and with iron deficiency (ID) being its leading cause [1]. ID is highly prevalent among women of reproductive age, affecting 1 in 4 women globally and 1 in 5 women in the United Kingdom [2,3]. During pregnancy, ID is linked to serious outcomes such as postpartum hemorrhage, preterm birth, maternal death, stillbirth, neonatal anemia, and impaired infant neurodevelopment [[4], [5], [6], [7], [8], [9]]. In the United Kingdom, anemia screening typically involves measuring hemoglobin concentrations at 12 and 28 gestational weeks (GW) [10]; however, this marker becomes abnormal only in the more advanced stages of deficiency. Because 85% of women of reproductive age with ID do not have anemia [2], most cases of ID at 12 GW may remain undiagnosed, leaving pregnant women with inadequate iron stores to meet the increased iron demands of pregnancy. The widespread prevalence of ID and the reliance on screening methods that only detect severe deficiency underscore the ineffectiveness of current approaches in identifying women at risk of iron deficiency anemia (IDA).

Obesity is the most common medical condition in women of reproductive age, affecting one-third of the global population and doubling in prevalence over the past 4 decades [11]. In the United Kingdom, 1 in 4 pregnancies are affected by obesity [12], a condition that substantially elevates risk of severe maternal complications, including thrombotic embolism, heart failure, and perinatal mortality. Moreover, obesity is associated with an increased likelihood of long-term cardiovascular and metabolic disorders in both the mother and her offspring [13,14].

Several studies have explored the relationship between obesity and iron status during pregnancy, with many reporting that women with obesity tend to have lower iron status, as indicated by markers such as transferrin saturation (TSAT) and serum iron concentrations [15,16]. However, results from studies comparing common clinical markers have been inconsistent [[15], [16], [17], [18], [19]]. Ferritin, which is widely used to diagnose ID, has high sensitivity in reflecting iron stores but does not reliably indicate iron status in individuals with inflammation, and may increase in the presence of inflammation [20]. Obesity is associated with chronic inflammation; thus, pregnant women with obesity who are iron deficient may be frequently undiagnosed due to elevated ferritin levels. This might result in untreated ID, exposing them to a greater risk of pregnancy-related complications. Some studies suggest that the soluble transferrin receptor (sTfR) may be increased in individuals with overweight or obesity, indicating a shift toward ID [15,21,22]. Despite this, the overall relationship between obesity and iron status remains unclear [[17], [18], [19],^23^], and there is an urgent need to improve ID screening policies, particularly for women with obesity, to ensure timely detection and treatment.

Although obesity is associated with a state of chronic inflammation, it is still unknown whether obesity-related inflammation directly influences iron metabolism. Inflammatory markers such as C-reactive protein (CRP) and IL-6 are elevated in women with overweight/obesity [15,18,21]. Although IL-6 has been proposed to stimulate the production of hepcidin, the key regulator of iron metabolism, other cytokines, such as IL-22, TNF-α, and IL-1, may also be involved [[24], [25], [26]]. Current knowledge on hepcidin indicates a typical decrease in the second and third trimesters to meet the increased iron demands during a healthy pregnancy [27]. However, pregnant women with overweight/obesity may have higher hepcidin concentrations than women of normal weight [15,16,22,23,28,29], which favors functional deficiency by decreasing the availability of circulating iron [30]. Although this theory may underlie the observed changes in iron markers in obesity, it has not been extensively researched [[17], [18], [19],^21^], leaving the impact of adiposity on iron metabolism and the possible mechanisms involved remain poorly understood, especially during pregnancy.

The alarmingly high prevalence of ID and obesity among pregnant women, along with the frequent underdiagnosis of ID, demands urgent action with a view to the implementation of more effective strategies for identifying women at risk of ID and anemia. Moreover, clarity is needed on the biological mechanisms that result in a lower iron status in pregnant women with higher adiposity. Therefore, this study aimed to investigate the association of maternal adiposity on iron status, explore the mediating role of inflammation, and evaluate how adiposity affects the predictive accuracy of early pregnancy iron markers to determine risk of late pregnancy ID.

## Methods

### Study design and data collection

This is a secondary analysis of a double-blinded randomized controlled intervention study (Impact of Maternal Body Weight on Vitamin D Status During Pregnancy, MO-VITD trial), with the original study details and full methodology reported by Alhomaid et al. [31] (registered at clinicaltrials.gov ID NCT02713009). In brief, the MO-VITD trial recruited healthy singleton pregnant women, in their first trimester, aged ≥18 y, and with BMI ≥ 18.5 kg/m^2^. Participants with diabetes mellitus, liver or renal chronic disease, autoimmune disorders, acute infections, chorioamnionitis, hematological disorders (other anemia causes), malabsorptive syndromes were excluded. Blood samples were taken at 12, 28, and 36 GW, and an umbilical cord sample after delivery. At these timepoints, anthropometric measurements (weight and height; BMI, and body composition by bioelectrical impedance; TANITA MC-780MA) were also taken. Participants were randomly assigned to receive either 10 μg or 20 μg vitamin D/d from 12 GW through to delivery. A total of 240 women were recruited to the study and were divided into 2 groups: 120 women received a multivitamin with 10 μg of vitamin D (placebo), and 120 women received a multivitamin with 20 μg of vitamin D. Each multivitamin contained 17 mg elemental iron. Health and lifestyle data were collected via questionnaire at 12 GW. At 28 GW, participants completed a 4-day food diary, and from this, daily dietary iron intake was estimated.

Hematological markers including hemoglobin, red cell blood count (RBC), mean cell volume (MCV), mean cell hemoglobin (MCH), red cell distribution width (RDW), and mean cell hemoglobin concentration (MCHC) from 12, 28 GW and maternal postpartum discharge after birth were measured with routine biochemical analyzers within the hospital laboratory and results were collected from maternal notes. Ethical approval was obtained for access to additional clinical data records, not collected as part of the original MO-VITD trial (23/NI/0087, REC reference number 321833, research governance number 23/0033).

### Laboratory analyses

To assess differences in iron and inflammatory status between mothers with different maternal adiposity and in iron transfer to their newborns, iron and inflammatory markers were measured in maternal blood samples as well as in umbilical cord blood samples. Stored serum samples from 12, 28, 36 GW and from umbilical cord blood were used to perform the analyses of iron and inflammatory markers: ferritin, transferrin, serum iron, hepcidin, sTfR, IL-6, TNF-α, IL-1 beta (IL-1β), and interferon gamma (IFN-γ). TSAT, unsaturated iron binding capacity (UIBC), and total iron binding capacity (TIBC) were calculated from transferrin and serum iron measurements (UIBC = TIBC-serum iron and TSAT = (serum iron/TIBC)∗100) [32]. Ferritin, serum iron, and transferrin analyses were performed at Causeway Hospital laboratory, Coleraine, Northern Ireland, using Elecsys Ferritin, cobas, Roche; IRON2, cobas, Roche; and TRSF2 Tina-quant Transferrin ver.2, cobas, Roche, respectively. Hepcidin, sTfR, and inflammatory markers (IFN-γ, IL1β, IL-6, and TNF-α) analyses were performed at the Nutrition Innovation Centre for Food and Health laboratory, Coleraine campus, Ulster University, using Quantikine TM ELISA, Human Hepcidin Immunoassay, R&D Systems; sTfR Human sTfR ELISA, BioVendor Group; and S-PLEX Platform, Proinflammatory Panel 1 (human) Kit, Meso Scale Diagnostics respectively. CRP results, already analyzed in blood serum for the original study (Meso Scale Diagnostics multiplex assay, lower limit detection of 3.0 pg/mL), were used for this analysis.

### Definitions

The sample population was divided into 3 groups according to BMI at 12 GW: normal weight 18.5–24.9 kg/m^2^, overweight 25.0–29.9 kg/m^2^, and obesity ≥30 kg/m^2^. ID was defined using serum ferritin and sTfR. ID was defined when ferritin was <15 μg/L [33,34]. In addition, ID was defined when sTfR was >1.513 μg/mL (97.5^th^ percentile as reported from Human sTfR ELISA BioVendor Group). Statistical analysis on IDA (defined as ferritin and hemoglobin below the reference cutoff) could not be performed, given the low number of participants with anemia in this analysis.

A modified version of a cytokine-based inflammation score [35] was used to categorize the sample population into high compared with low inflammation, dependent on the concentrations of proinflammatory cytokines measured at baseline. Cytokine concentrations (IL-6, TNF-α, IFN-γ, and IL-1β) were divided into tertiles, assigning a score; 1 point to tertile 1, 2 points to tertile 2, and 3 points to tertile 3. These points were then summed up to obtain the inflammation score. The inflammation score was divided into high or low inflammation based on the 50th centile (high inflammation score ≥8, low inflammation score <8).

### Statistical methods

Normality was checked using Kolmogorov–Smirnov test. Continuous variables were described with mean and SD, and categorical variables with absolute frequency and percentage.

Hematological markers at 12, 28 GW, and postpartum discharge, iron and inflammatory markers at 12, 28, 36 GW, and in umbilical cord blood were the outcomes. Ferritin was adjusted for inflammation using CRP concentrations applying internal regression correction [36,37]. Adiposity measures, BMI, fat mass, visceral fat, and fat mass index (FMI; calculated using the formula: fat mass (kg)/height^2^), were considered independent variables.

Age, smoking status, educational level, and vitamin D intervention group were considered covariates. We performed multiple imputation to address missing data, as the proportion of missing values exceeded 5%. Statistical analyses on the imputed datasets followed Rubin's Rules. A total of 5 imputations were conducted, and the resulting pooled estimates and *P* values from each were recorded.

Means of each marker at different timepoints were compared between BMI groups using analysis of covariance repeated measures, and Tukey post-hoc tests, and general linear model adjusting for covariates for comparisons at each timepoint and according to the inflammation score (high and low). Linear regression analyses were performed between hematological/iron markers and adiposity measures at different timepoints using separate multiple linear regression models. Each iron marker was considered a dependent variable in separate models. Each model included a dependent variable, one of the adiposity measures as an independent variable (BMI, fat mass, visceral fat, and FMI), along with covariates (age, smoking status, education level, and vitamin D intervention group). Model fit was assessed using the coefficient of determination (*r*^2^ and adjusted *r*^2^). Normality, homoscedasticity, and independence of residuals were evaluated. Collinearity was assessed with the variation inflation factor, which resulted in <5 in all the analyses (low collinearity). Interaction terms were created using the inflammation score with each adiposity measure to evaluate a possible mediating effect of inflammation on hematological marker concentrations. The prevalence of ID at 12 and 36 GW, defined by ferritin <15 μg/L, and sTfR>1.513 μg/mL, was compared between adiposity groups using Chi^2^ test.

Logistic regression analyses were performed to assess the association between adiposity measures and ID at 12 and 36 GW. ID at 12 and 36 GW was considered the dependent variable in separate models. Adiposity measures were considered independent variables, and covariates were age, smoking status, education level, and vitamin D intervention group. Each model included a dependent variable, an independent adiposity variable, and all covariates.

Both linear and logistic regression analyses were corrected for multiple comparisons using the Benjamini-Hochberg test.

Receiver operating characteristic (ROC) analysis was performed to evaluate the performance of each marker at 12 GW in predicting ID at 36 GW for the overall sample and separately for those of normal weight and overweight/obesity. The ROC curve was generated by plotting the true positive rate (sensitivity) against the false positive rate (1-specificity) at various threshold settings. The AUC was computed as a summary metric of model performance. Sensitivity, specificity, positive predictive value, and negative predictive value were calculated.

Statistical analyses were performed using SPSS (Statistical Package for the Social Sciences software, version 29; IBM). Results were considered significant at *P* < 0.05.

## Results

**FIGURE 1:**
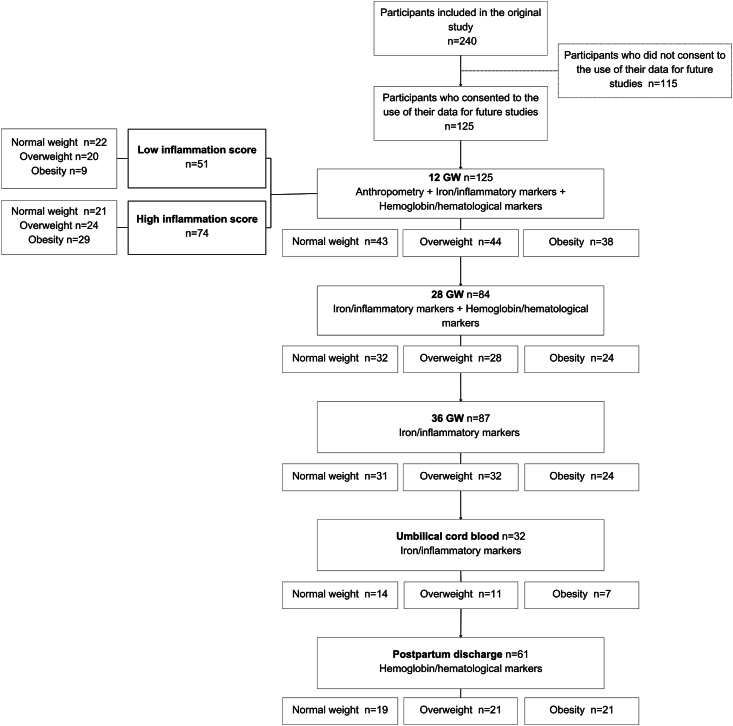
Flowchart showing the number of participants included according to BMI groups at each timepoint. High and low inflammation at 12 GW defined as follows: high inflammation: score ≥8, low inflammation: score <8. Normal weight: BMI 18.5–24.9 kg/m^2^, overweight: BMI 25.0–29.9 kg/m^2^, obesity: BMI ≥30 kg/m^2^. GW, gestational weeks.

Of 240 pregnant women recruited for the original MO-VITD trial, 125 consented to their data being used for future studies and therefore included in our analysis (data available at 12 GW: normal weight *n* = 43, overweight *n* = 44, obesity *n* = 38) (Figure 1). Data from 84 participants were available at 28 GW, *n* = 87 at 36 GW, *n* = 32 umbilical cord blood samples, and *n* = 61 participants with available hematologic marker results at hospital discharge, after delivery. Of the 125 included, women who discontinued the study (between 12 GW and 28 GW) had a younger maternal age at the time of booking (28.4 ± 4.8 vs 30.5 ± 5.2 y, *P* = 0.035) compared with the group with complete data. However, no other differences were observed in the general characteristics, obstetric and neonatal outcomes, history of anemia/transfusions, iron supplementation at booking, and hematologic/iron markers concentrations at booking.

Table 1 shows the baseline maternal and infant characteristics included in the analysis. The educational level was lower in pregnant women with obesity and overweight compared with normal weight (*P* = 0.040). Compliance with the original intervention trial (10 μg/L or 20 μg/L of vitamin D plus a multivitamin) was excellent and similar across the BMI groups (normal weight 91.7%, overweight 86.9%, obesity 90.7%; *P* = 0.328). No differences were observed in the history of anemia in previous pregnancies, nor in the percentage of pregnant women receiving iron supplementation at the beginning of the pregnancy. No differences were observed in maternal or neonatal outcomes or in the mean concentrations of hemoglobin, MCV, MCH, RDW, and MCHC at maternal postpartum discharge, except for RBC, which was lower in the overweight compared with the normal weight group (3.54 vs 3.79 10^12^/L, *P* = 0.007). The overall mean iron dietary intake was 12.5 ± 6.1 mg/d. Due to the low number of pregnant women with obesity who completed the 4-day food diary, we were unable to report meaningful statistical comparisons on dietary iron intake between BMI groups.

**TABLE 1:** Maternal and infant characteristics of the included participants.

	Normal weight (*n* = 43)	SD/%	Overweight (*n* = 44)	SD/%	Obesity (*n* = 38)	SD/%
Maternal age (y)	31.0	5.1	29.2	5.0	29.3	5.2
Weight (kg)	61.4^a^	6.7	73.5^b^	6.5	91.2^c^	13.8
Height (m)	1.60	0.06	1.63	0.07	1.63	0.06
BMI (kg/m^2^)	22.8^a^	1.6	27.6^b^	1.4	34.2^c^	4.2
At least secondary school, *n* (%)	8∗	18.6	12∗	27.3	16∗	44.4
Third level education or above, *n* (%)	35∗	81.4	32∗	72.7	20∗	55.6
Primigravida, *n* (%)	11	25.6	21	47.7	18	48.0
Previous miscarriage, *n* (%)	15	34.9	12	27.2	10	26.3
Non-smoking at enrollment, *n* (%)	41	95.3	40	90.9	32	86.5
Systolic BP (mm Hg)	117.0	12.3	120.9	10.5	122.9	11.4
Diastolic BP (mm Hg)	69.8^a^	9.3	73.9^b^	7.6	73.5^ab^	7.9
Iron supplementation at booking, *n* (%)	20	46.5	19	43.2	13	34.2
Maternal outcomes						
GW delivery (weeks)	39.5	1.7	39.8	1.2	39.5	2.3
Vaginal delivery, *n* (%)	24	55.8	22	50.0	19	50.0
C-section, *n* (%)	14	32.5	13	29.5	15	39.4
Maternal blood loss (mL)	461.1	304.3	568.1	386.4	537.2	357.7
Hematological markers at discharge						
Hemoglobin (g/L)	110.9	11.5	106.7	11.8	107.9	11.0
RBC (10^12^/L)	3.79^a^	0.5	3.54^b^	0.5	3.73^ab^	0.5
MCV (fL)	87.8	4.0	88.1	4.5	86.8	3.5
MCH (pg)	29.6	2.0	29.9	2.0	29.5	1.8
MCHC (g/L)	338.6	8.6	339.2	7.0	337.0	13.3
RDW (%)	14.3	1.4	14.3	1.1	14.3	1.4
Neonatal outcomes						
Apgar 1 (min)	8.4	1.1	8.7	0.6	8.2	1.5
Apgar 5 (min)	8.9	0.6	8.9	0.4	8.6	1.4
Female, *n* (%)	19	44.1	24	54.5	23	60.5
Birthweight (g)	3588	537	3540	359	3566	373
Length (cm)	52.7	4.1	52.6	2.6	52.5	3.1
Head circumference (cm)	35.7	3.5	35.1	1.5	35.1	1.4

Data presented as mean and SD or absolute number and percentage. For numeric variables *P* value obtained from one-way ANOVA with Tukey post-hoc tests. Different superscript letters denote statistical difference between groups in continuous variables. Log-transformed variables as appropriate. For categorical variables *P* values obtained with Chi^2^ test.

Abbreviations: ANOVA, analysis of variance; BP, blood pressure; C-section, cesarean section; GW, gestational weeks; MCH, mean cell hemoglobin; MCHC, mean cell hemoglobin concentration; MCV, mean cell volume; RBC, red blood cell count; RDW, red cell distribution width.

∗ Denotes a significant difference in categorical variables.

**FIGURE 2:**
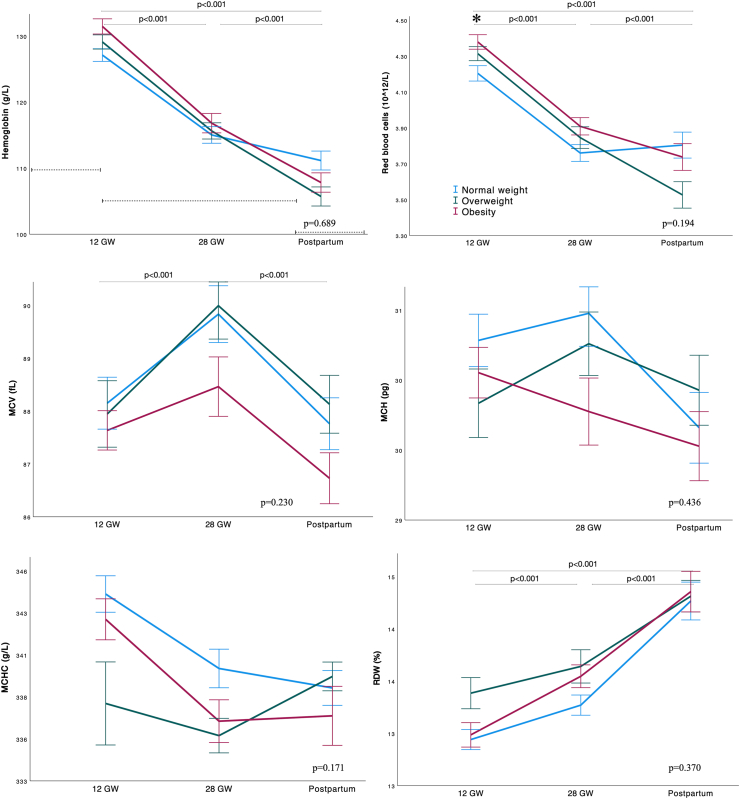
Changes in mean hematological markers throughout pregnancy according to BMI group. Line graphs showing mean and SE. Normal weight: BMI 18.5–24.9 kg/m^2^, overweight: BMI 25.0–29.9 kg/m^2^, obesity: BMI ≥30 kg/m^2^. Repeated measures ANCOVA with Tukey's post-hoc test used to compare differences between timepoints, and general linear model applied for comparisons at each timepoint, adjusting for maternal age, smoking, educational status, and vitamin D intervention group. Special characters denote statistical difference between BMI groups at each timepoint. Special characters next to *P* values denote temporal differences between BMI categories: ∗normal weight vs. obesity; †normal weight vs. overweight; ‡*P* overweight vs. obesity. *P* values at the top of the graph show overall significance between timepoints. ANCOVA, analysis of covariance; GW, gestational weeks; MCH, mean cell hemoglobin; MCHC, mean cell hemoglobin concentrations; MCV, mean cell volume; RDW, red cell distribution width.

**FIGURE 3:**
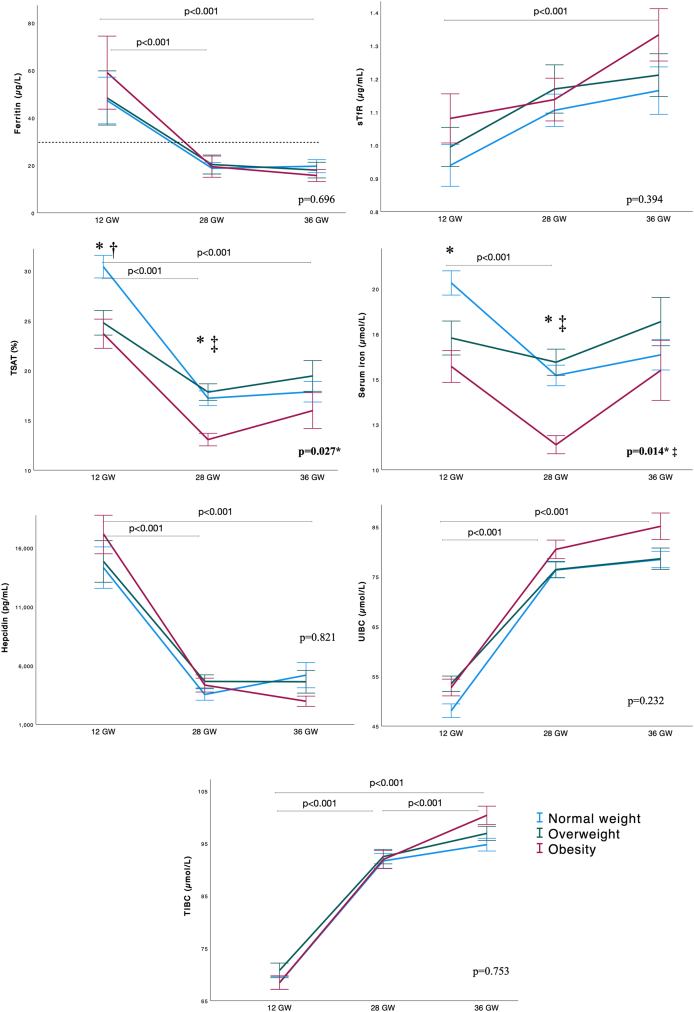
Changes in mean iron markers throughout pregnancy according to BMI group. Line graphs showing mean and SE. Normal weight: BMI 18.5–24.9 kg/m^2^, overweight: BMI 25.0–29.9 kg/m^2^, obesity: BMI ≥30 kg/m^2^. Repeated measures ANCOVA with Tukey's post-hoc test used to compare differences between timepoints, and general linear model applied for comparisons at each timepoint, adjusting for maternal age, smoking, educational status, and vitamin D intervention group. Special characters denote statistical difference between BMI groups at each timepoint. Special characters next to *P* values denote temporal differences between BMI categories: ∗normal weight vs. obesity. † normal weight vs. overweight. ‡ overweight vs. obesity. *P* values at the top of the graph show overall significance between timepoints. ANCOVA, analysis of covariance; GW, gestational weeks; sTfR, soluble transferrin receptor; TIBC, total iron binding capacity; TSAT, transferrin saturation; UIBC, unsaturated iron binding capacity.

FIGURE 2, FIGURE 3 present the mean concentrations of hematological markers at 12, 28 GW and postpartum discharge, and iron markers at 12, 28, and 36 GW, stratified by BMI. At 12 GW, RBC was higher in pregnant women with obesity compared with normal weight (4.38 vs 4.20 10^12^/L; *P* = 0.047). Women with obesity had lower TSAT and serum iron than those of normal weight at both 12 and 28 GW (for obesity, normal weight, respectively: TSAT 12 GW 23.9 vs 30.3%, *P* = 0.016, 28 GW 13.2 vs 17.2%, *P* < 0.001; serum iron 12 GW 15.8 vs 20.6 μmol/L, *P* = 0.022; 28 GW 11.5 vs 15.1 μmol/L, *P* < 0.001). At 36 GW, there were no overall differences in ferritin concentrations between BMI groups. However, among women with a high inflammation score, those with obesity had lower ferritin levels at 36 GW compared with those of normal weight (TABLE 2, TABLE 3). There were no observed differences in fetal iron markers between BMI groups.

**TABLE 2:** Mean concentrations of hematological and iron markers at different timepoints of pregnancy according to BMI at 12 GW in women with low inflammation score.

	Normal weight (*n* = 22)		Overweight (*n* = 20)		Obesity (*n* = 9)		
12 GW	Mean	SD	Mean	SD	Mean	SD	*P* value

Hemoglobin (g/L)	127.6	7.3	130.1	8.0	133.5	8.6	0.171
RBC (10^12^/L)	4.23	0.3	4.38	0.2	4.42	0.3	0.133
MCV (fL)	88.2	4.3	88.1	4.2	87.7	2.8	0.810
MCH (pg)	30.1	1.6	29.5	1.6	30.5	1.1	0.311
MCHC (g/L)	342.6	7.3	340.6	10.8	346.9	8.7	0.277
RDW (%)	13.1^ab^	0.6	13.4^a^	1.0	12.6^b^	0.7	0.085
Ferritin (μg/L)	44.1^a^	25.5	45.0^ab^	34.0	83.8^b^	69.5	0.126
sTfR (μg/mL)	1.03	0.6	0.99	0.5	1.02	0.4	0.963
TSAT (%)	30.6	7.4	26.0	8.8	26.2	11.4	0.292
Serum iron (μmol/L)	20.8	4.8	17.6	6.4	16.9	6.9	0.212
Hepcidin (pg/mL)	13435	11889	13394	15531	18828	13561	0.240
UIBC (μmol/L)	48.3	8.9	51.2	12.0	48.3	9.0	0.675
TIBC (μmol/L)	69.1	7.7	68.9	10.6	65.2	5.2	0.640

28 GW	Mean	SD	Mean	SD	Mean	SD	*P* value

Hemoglobin (g/L)	117.7	8.7	117.9	8.8	115.4	8.4	0.709
RBC, 10^12^/L	3.84	0.3	3.87	0.4	3.83	0.3	0.374
MCV (fL)	90.2	3.9	90.0	3.6	89.0	4.2	0.466
MCH (pg)	30.4	1.4	30.4	1.3	30.1	1.6	0.502
MCHC (g/L)	338.6	6.7	337.0	7.1	338.2	5.8	0.641
RDW (%)	13.3	0.7	13.6	1.3	13.3	0.6	0.465
Ferritin (μg/L)	18.0	8.9	20.2	18.9	21.3	13.6	0.681
sTfR (μg/mL)	1.13	0.3	1.21	0.7	1.30	0.5	0.493
TSAT (%)	15.7^ab^	4.8	19.0^a^	7.6	13.5^b^	4.5	0.038
Serum iron (μmol/L)	13.8^ab^	3.5	16.9^a^	6.6	12.2^b^	4.7	0.042
Hepcidin (pg/mL)	3381	3703	3901	3778	5363	5047	0.611
UIBC (μmol/L)	77.7	13.4	76.5	14.9	80.1	11.7	0.582
TIBC (μmol/L)	92.0	12.3	93.2	12.4	93.1	12.4	0.934

36 GW	Mean	SD	Mean	SD	Mean	SD	*P* value

Ferritin (μg/L)	19.1	10.3	19.9	15.2	18.2	9.8	0.824
sTfR (μg/mL)	1.27	0.6	1.18	0.5	1.30	0.6	0.710
TSAT (%)	18.3	10.0	20.2	15.7	13.8	6.2	0.386
Serum iron (μmol/L)	16.7	7.9	19.5	13.9	13.6	5.8	0.369
Hepcidin (pg/mL)	5029	10600	3597	4169	3089	2452	0.563
UIBC (μmol/L)	78.9	15.7	77.5	18.8	85.5	15.2	0.257
TIBC (μmol/L)	95.6	10.7	96.9	10.7	98.4	13.4	0.374

High and low inflammation at 12 GW was defined as follows: high inflammation: score ≥8, low inflammation: score <8. Data presented as mean and SD. *P* value obtained from ANCOVA and Tukey post-hoc tests, adjusted for maternal age, smoking, educational status, and vitamin D intervention arm. Different superscript letters denote statistical difference between groups. Log-transformed variables as appropriate.

Abbreviations: ANCOVA, analysis of covariance; GW, gestational weeks; MCH, mean cell hemoglobin; MCHC, mean cell hemoglobin concentration; MCV, mean cell volume; RBC, red blood cell count; RDW, red cell distribution width; sTfR, soluble transferrin receptor; TIBC, total iron binding capacity; TSAT, transferrin saturation; UIBC, unsaturated iron binding capacity.

**TABLE 3:** Mean concentrations of hematological and iron markers at different timepoints of pregnancy according to BMI at 12 GW in women with high inflammation score.

	Normal weight (*n* = 21)		Overweight (*n* = 24)		Obesity (*n* = 29)		
12 GW	Mean	SD	Mean	SD	Mean	SD	*P* value

Hemoglobin (g/L)	126.4	7.9	128.1	8.9	130.6	8.5	0.403
RBC (10^12^/L)	4.17	0.3	4.27	0.3	4.36	0.3	0.167
MCV (fL)	88.1	3.3	88.2	5.5	87.6	2.7	0.747
MCH (pg)	30.4	1.2	30.0	2.1	29.9	1.3	0.464
MCHC (g/L)	345.4	9.1	335.4	23.2	341.2	8.7	0.079
RDW (%)	12.8	0.8	13.3	1.3	13.1	0.8	0.239
Ferritin (μg/L)	50.5	37.9	51.1	40.7	51.3	35.3	0.948
sTfR (μg/mL)	0.83	0.2	0.98	0.4	1.10	0.6	0.254
TSAT (%)	30.1	10.5	23.3	10.6	23.2	11.2	0.213
Serum iron (μmol/L)	19.7	5.9	16.6	8.3	15.5	6.7	0.377
Hepcidin (pg/mL)	15667	16919	16004	13460	17152	12728	0.437
UIBC (μmol/L)	47.7	12.8	55.5	12.5	54.0	13.5	0.245
TIBC (μmol/L)	67.4	11.8	72.2	11.6	69.5	10.5	0.402

28 GW	Mean	SD	Mean	SD	Mean	SD	*P* value

Hemoglobin (g/L)	112.4	10.7	113.9	10.2	117.4	11.7	0.157
RBC (10^12^/L)	3.69	0.3	3.84	0.5	3.93	0.3	0.087
MCV (fL)	89.5	4.4	89.7	6.0	88.3	4.0	0.468
MCH (pg)	30.5	2.1	30.0	2.1	29.7	1.7	0.492
MCHC (g/L)	340.8	10.7	334.7	8.9	336.0	10.3	0.207
RDW (%)	13.2	0.6	13.6	1.2	13.6	0.8	0.224
Ferritin (μg/L)	19.7	7.1	19.7	11.9	18.8	14.5	0.501
sTfR (μg/mL)	1.08	0.4	1.12	0.4	1.08	0.4	0.604
TSAT (%)	18.7^a^	6.6	16.5^a^	5.8	13.1^b^	4.7	0.019
Serum iron (μmol/L)	16.5^a^	5.1	14.8^a^	5.0	11.3^b^	3.3	<0.001
Hepcidin (pg/mL)	3713	4184	5322	5302	3995	4300	0.187
UIBC (μmol/L)	74.7	12.3	76.6	12.4	80.2	14.9	0.391
TIBC (μmol/L)	90.9	10.4	91.8	10.6	91.4	13.5	0.926

36 GW	Mean	SD	Mean	SD	Mean	SD	*P* value

Ferritin (μg/L)	20.3^a^	7.9	16.6^ab^	5.8	15.0^b^	7.4	0.041
sTfR (μg/mL)	1.04	0.4	1.22	0.5	1.32	0.6	0.245
TSAT (%)	17.5	6.1	18.7	9.3	16.8	15.1	0.871
Serum iron (μmol/L)	16.1	5.2	17.0	7.2	16.3	15.1	0.938
Hepcidin (pg/mL)	5415	6209	5398	9865	3049	3527	0.871
UIBC (μmol/L)	77.9	10.1	79.4	16.4	84.6	21.6	0.575
TIBC (μmol/L)	94.0	8.8	96.3	11.4	100.8	13.3	0.306

High and low inflammation at 12 GW defined as follows: high inflammation: score ≥8, low inflammation: score <8. Data presented as mean and SD. *P* value obtained from one-way ANCOVA and Tukey post-hoc tests, adjusted for maternal age, smoking, educational status, and vitamin D intervention arm. Different superscript letters denote statistical difference between groups. Log-transformed variables as appropriate.

Abbreviations: ANCOVA, analysis of covariance; GW, gestational weeks; MCH, mean cell hemoglobin; MCHC, mean cell hemoglobin concentration; MCV, mean cell volume; RBC, red blood cell count; RDW, red cell distribution width; sTfR, soluble transferrin receptor; TIBC, total iron binding capacity; TSAT, transferrin saturation; UIBC, unsaturated iron binding capacity.

**FIGURE 4:**
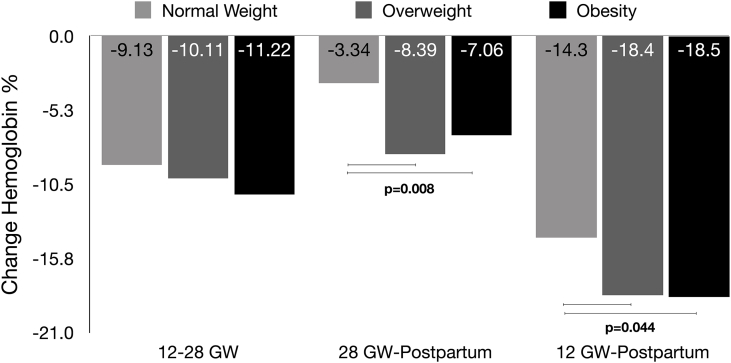
Percentage of change of hemoglobin across different timepoints of pregnancy (between 12 and 28 GW, between 28 GW and postpartum discharge, and between 12 GW and postpartum discharge) according to BMI group. General linear model and Tukey post-hoc test performed adjusting for maternal age, smoking, educational status, and vitamin D intervention group. Normal weight: BMI 18.5–24.9 kg/m^2^, overweight: BMI 25.0–29.9 kg/m^2^, obesity: BMI ≥30 kg/m^2^. GW, gestational weeks.

Examining biomarkers across pregnancy, hemoglobin and RBC concentrations significantly decreased from 12 GW to postpartum discharge (*P* < 0.001). MCV increased between 12 and 28 GW, followed by a decline from 28 GW to postpartum discharge. In contrast, RDW showed a consistent increase from 12 GW to postpartum discharge (Figure 2). No changes were observed in MCH and MCHC. Furthermore, there were no interactions between BMI groups and time for any hematological parameter, with the exception of hemoglobin. Hemoglobin concentrations declined more markedly in women with overweight and obesity compared with those of normal weight between 12 GW and postpartum discharge (obesity: −18.5%, overweight: −18.4%, normal weight: −14.3%; *P* = 0.044) (Figure 4).

Between 12 and 28 GW, concentrations of ferritin, TSAT, serum iron, and hepcidin declined, whereas levels of sTfR, UIBC, and TIBC increased markedly. From 28 to 36 GW, these markers remained relatively stable, except for TIBC, which continued to increase through the end of pregnancy (Figure 3). No differences across pregnancy were observed between BMI groups except for TSAT and serum iron, which were consistently lower in women with obesity compared with those with normal weight (Figure 3).

**FIGURE 5:**
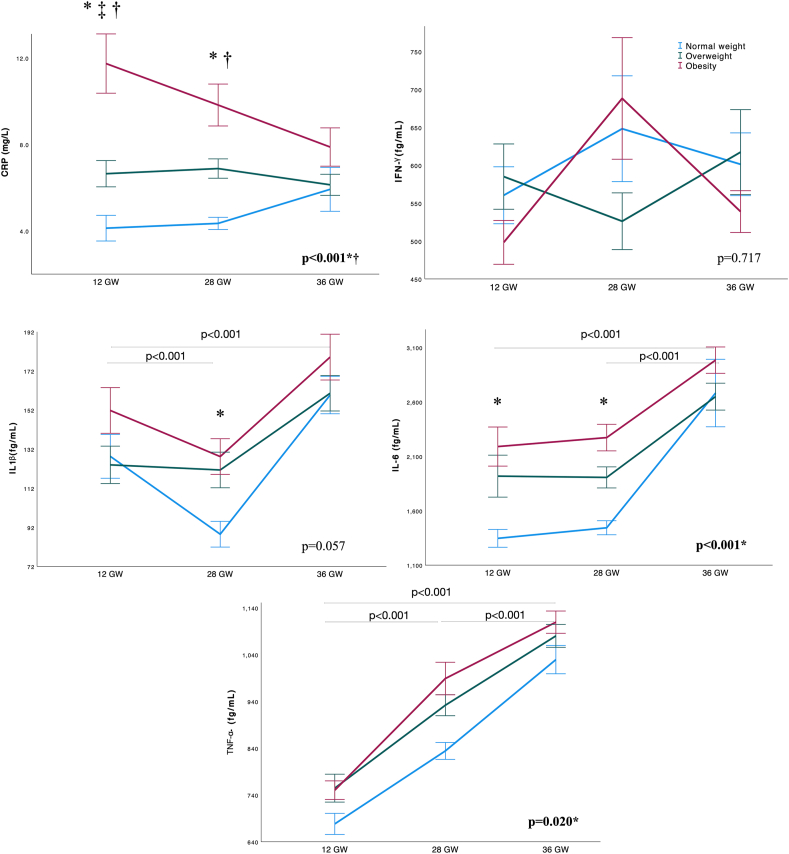
Changes in mean inflammatory biomarkers throughout pregnancy according to BMI group. Line graphs showing mean and SE. Normal weight: BMI 18.5–24.9 kg/m^2^, overweight: BMI 25.0–29.9 kg/m^2^, obesity: BMI ≥30 kg/m^2^. Repeated measures ANCOVA with Tukey’s post-hoc test used to compare differences between timepoints, and general linear model applied for comparisons at each timepoint, adjusting for maternal age, smoking, educational status, and vitamin D intervention group. Special characters denote statistical difference between BMI groups at each timepoint. Special characters next to *P* values denote temporal differences between BMI categories. ∗ normal weight vs. obesity. † normal weight vs. overweight. ‡ overweight vs. obesity. *P* values at the top of the graph showing overall significance between timepoints. ANCOVA, analysis of covariance; CRP, C-reactive protein; GW, gestational weeks; IFN-γ, interferon gamma; IL-1β, interleukin-1 beta; IL-6, interleukin-6; TNF-α, Tumor necrosis factor α.

Figure 5 shows the mean CRP, IFN-γ, IL-6, IL-1β, and TNF-α concentrations at 12, 28, and 36 GW according to BMI. CRP concentrations at 12, 28 GW increased with higher BMI (for normal weight, overweight, and obesity, respectively: 12 GW 4.12 vs 6.68 vs 11.7 mg/L, *P* < 0.001; 28 GW 4.45 vs 6.87 vs 9.70 mg/L, *P* < 0.001). At 12 and 28 GW, women with obesity had higher IL-6 concentrations compared with normal weight women (12 GW 2169 vs 1371 fg/mL, *P* = 0.004; 28 GW 2251 vs 1460 fg/mL, *P* = 0.021 in obesity and normal weight, respectively). TNF-α and IFN-γ levels did not differ between BMI groups at any timepoint. However, IL-1β concentrations were higher in women with obesity compared with those of normal weight at 28 GW (127.1 vs 89.4 fg/mL, *P* = 0.038).

In multiple linear regression, BMI and FMI were positively associated with hemoglobin, all adiposity measures positively associated with UIBC, and negatively associated with TSAT and serum iron at 12 GW (Table 4). Higher measures of adiposity at 12 GW predicted lower ferritin concentrations (BMI *β* = –0.253, *P* = 0.020, and FMI *β* = –0.265, *P* = 0.010) and higher sTfR (particularly fat mass and visceral fat *β* = 0.276, *P* = 0.044, *β* = 0.253, *P* = 0.033) at 36 GW (Table 5). A significant interaction effect of the inflammation score was observed in the association between adiposity and hemoglobin and RBC at 12 GW (hemoglobin: BMI∗inflammation score *β* = –0.310, *P* = 0.023; fat mass∗inflammation score *β* = –0.467, *P* = 0.025; visceral fat∗inflammation score *β* = –0.667, *P* = 0.024; FMI∗inflammation score *β* = –0.456, *P* = 0.036).

**TABLE 4:** Association between adiposity measures and hematological and iron markers at 12 GW.

	BMI (kg/m^2^)	Fat mass (kg)	Visceral fat (cm^2^)	FMI (kg/m^2^)
Hemoglobin (g/L)				
Standardized coefficient *β*	0.208	0.224	0.182	0.211
*P* value	0.026	0.016	0.051	0.023
Corrected *P* value^1^	0.034	0.064	0.051	0.043
RBC (10^12^/L)				
Standardized coefficient *β*	0.206	0.237	0.187	0.196
*P* value	0.054	0.020	0.083	0.069
Corrected *P* value^1^	0.108	0.080	0.083	0.092
MCV (fL)				
Standardized coefficient *β*	–0.029	–0.045	–0.044	–0.031
*P* value	0.765	0.658	0.662	0.766
Corrected *P* value^1^	1.000	1.000	1.000	0.766
MCH (fL)				
Standardized coefficient *β*	–0.052	–0.086	–0.068	–0.060
*P* value	0.593	0.371	0.484	0.541
Corrected *P* value^1^	0.593	1.000	0.968	0.721
MCHC (g/L)				
Standardized coefficient *β*	–0.077	–0.106	–0.078	–0.081
*P* value	0.398	0.259	0.402	0.388
Corrected *P* value^1^	0.530	1.000	0.402	0.776
RDW (%)				
Standardized coefficient *β*	0.068	0.098	0.092	0.076
*P* value	0.456	0.305	0.334	0.800
Corrected *P* value^1^	0.608	1.000	0.668	0.800
sTfR (μg/mL)				
Standardized coefficient *β*	0.143	0.185	0.200	0.163
*P* value	0.057	0.050	0.030	0.078
Corrected *P* value^1^	0.076	0.100	0.120	0.078
UIBC (μmol/L)				
Standardized coefficient *β*	0.231	0.210	0.226	0.236
*P* value	0.010	0.025	0.014	0.010
Corrected *P* value^1^	0.020	0.025	0.018	0.040
TIBC (μmol/L)				
Standardized coefficient *β*	0.086	0.020	0.047	0.060
*P* value	0.341	0.838	0.612	0.642
Corrected *P* value^1^	1.000	0.838	1.000	0.856
TSAT (%)				
Standardized coefficient *β*	–0.305	–0.343	–0.340	–0.340
*P* value	<0.001	<0.001	<0.001	<0.001
Corrected *P* value^1^	<0.001	<0.001	<0.001	<0.001
Serum iron (μmol/L)				
Standardized coefficient *β*	–0.284	–0.346	–0.334	–0.333
*P* value	0.001	<0.001	<0.001	<0.001
Corrected *P* value^1^	0.004	<0.001	<0.001	<0.001
Ferritin (μg/L)				
Standardized coefficient *β*	0.015	–0.023	0.003	–0.017
*P* value	0.879	0.792	0.985	0.853
Corrected *P* value^1^	1.000	1.000	0.985	1.000
Hepcidin (pg/mL)				
Standardized coefficient *β*	0.065	0.037	0.056	0.017
*P* value	0.497	0.402	0.554	0.183
Corrected *P* value^1^	0.662	0.804	0.554	0.732

Multiple linear regression analysis adjusted for age, smoking status, and educational status.

Abbreviations: FMI, fat mass index; GW, gestational weeks; MCH, mean cell hemoglobin; MCHC, mean cell hemoglobin concentration; MCV, mean cell volume; RBC, red blood cell count; RDW, red cell distribution width; sTfR, soluble transferrin receptor; TIBC, total iron binding capacity; TSAT, transferrin saturation; UIBC, unsaturated iron binding capacity.

1 *P* value was corrected for multiple comparisons using the Benjamini-Hochberg test.

**TABLE 5:** Association between adiposity measures at 12 GW and iron markers at 36 GW.

	BMI (kg/m^2^)	Fat mass (kg)	Visceral fat (cm^2^)	FMI (kg/m^2^)
Ferritin (μg/L)				
Standardized coefficient *β*	–0.253	–0.210	–0.243	–0.265
*P* value	0.015	0.068	0.035	0.010
Corrected *P* value^1^	0.020	0.272	0.070	0.010
TSAT (%)				
Standardized coefficient *β*	–0.021	0.002	0.016	0.000
*P* value	0.809	0.951	0.899	0.977
Corrected *P* value^1^	1.000	1.000	1.000	0.977
Serum iron (μmol/L)				
Standardized coefficient *β*	0.002	0.011	0.034	0.018
*P* value	0.982	0.918	0.735	0.872
Corrected *P* value^1^	0.982	1.000	1.000	1.000
Hepcidin (pg/mL)				
Standardized coefficient *β*	–0.154	–0.139	–0.143	–0.155
*P* value	0.147	0.231	0.171	0.163
Corrected *P* value^1^	0.588	0.231	0.228	0.326
UIBC (μmol/L)				
Standardized coefficient *β*	0.138	0.071	0.081	0.109
*P* value	0.142	0.448	0.378	0.245
Corrected *P* value^1^	0.568	0.448	0.504	0.490
TIBC (μmol/L)				
Standardized coefficient *β*	0.198	0.109	0.144	0.173
*P* value	0.035	0.253	0.128	0.070
Corrected *P* value^1^	0.140	0.253	0.170	0.140
sTfR (μg/mL)				
Standardized coefficient *β*	0.240	0.276	0.253	0.245
*P* value	0.040	0.011	0.025	0.022
Corrected *P* value^1^	0.040	0.044	0.033	0.044

Multiple linear regression analysis adjusted for age, smoking status, educational level, and vitamin D intervention group.

Abbreviations: FMI, fat mass index; GW, gestational weeks; sTfR, soluble transferrin receptor; TIBC, total iron binding capacity; TSAT, transferrin saturation; UIBC, unsaturated iron binding capacity.

1 *P* value was corrected for multiple comparisons using the Benjamini-Hochberg test.

**FIGURE 6:**
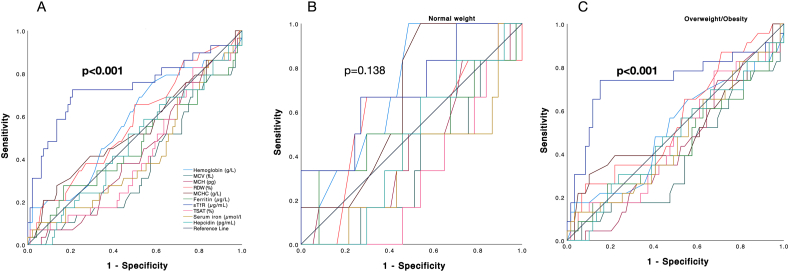
Accuracy of iron markers at 12 GW for the prediction of iron deficiency at 36 GW defined by sTfR. Receiver operating characteristic (ROC) analysis: (A) all samples. (B) Normal weight (BMI 18.5–24.9 kg/m^2^). (C) Overweight/obesity (BMI ≥ 25 kg/m^2^). Iron deficiency at 36 GW defined as sTfR>1.513 μg/mL. *P* < 0.05 considered significant. GW, gestational weeks; MCH, mean cell hemoglobin; MCHC, mean hemoglobin concentration; MCV, mean cell volume; RDW, red cell distribution width; sTfR, soluble transferrin receptor; TSAT, transferrin saturation.

The prevalence of anemia was 0.8%, 10.4%, and 16.8% at 12, 28 GW, and at postpartum discharge, respectively. At 12 GW, the prevalence of ID defined as ferritin <15 μg/L was 8.0% (normal weight 6.9%, overweight 9.1%, obesity 7.9%, *P* = 0.936). At 36 GW, ID prevalence defined as ferritin <15 μg/L was 40.0% (normal weight 30.2%, overweight 40.9%, and obesity 50.0%, *P* = 0.060). When ID was defined by sTfR, ID prevalence at 12 GW was 12.0% (normal weight 6.9%, overweight 11.3% and obesity 18.4%, *P* = 0.258) and at 36 GW 24.8% (normal weight 16.3%, overweight 22.7% and obesity 34.2%, *P* = 0.091). Multiple logistic regression analysis showed that higher BMI and adiposity measures at 12 GW predicted ID when defined as ferritin <15 μg/L at 36 GW (BMI: adjusted odds ratio (aOR) = 1.127, *P* = 0.007, fat mass aOR = 1.091, *P* = 0.025, visceral fat aOR = 1.265, *P* = 0.012, FMI: aOR = 1.183, *P* = 0.009); and also when ID was defined as sTfR>1.513 μg/mL at 12, and 36 GW (12 GW BMI aOR = 1.139, *P* = 0.010, fat mass aOR = 1.054, *P* = 0.038, visceral fat aOR = 1.302, *P* = 0.011, FMI aOR = 1.172, *P* = 0.026; 36 GW BMI aOR = 1.111, *P* = 0.039, fat mass aOR = 1.148, *P* = 0.019, visceral fat aOR = 1.267, *P* = 0.027, FMI aOR = 1.161, *P* = 0.028). ROC analysis showed that sTfR had the best accuracy in the prediction of ID at 36 GW (Figure 6). The cutoff of 1.018 μg/mL had the best performance in predicting ID categorized by sTfR at 36 GW with an AUC of 0.738 [95% confidence interval (CI): 0.62, 0.86; *P* < 0.001]. When this analysis was performed by BMI subgroups (normal weight compared with overweight/obesity), the AUC was 0.708 (95% CI: 0.47, 0.93; *P* = 0.138) and 0.744 (95% CI: 0.61, 0.88; *P* < 0.001), respectively. Using a cutoff point of 1.018 μg/mL, the sensitivity, specificity, positive predictive value, and negative predictive value were: 67.0%, 77.9%, 48.8%, and 88.1% for the entire sample, 57.1%, 72.2%, 28.6%, and 89.6% for pregnant women of normal weight, and 66.7%, 81.0%, 59.2%, and 85.4% for those with overweight/obesity.

## Discussion

This study found that despite daily iron supplementation of 17 mg, 40% of pregnant women developed ID in late pregnancy, with a significantly higher risk among women with obesity. Our findings show that higher levels of adiposity in the first trimester are linked with a more pronounced decline in hemoglobin concentrations later in pregnancy, as well as lower ferritin concentrations in women with obesity and elevated inflammation. Additionally, proinflammatory cytokines were found to negatively affect hemoglobin concentrations as early as 12 GW in women with higher adiposity. Hemoglobin and ferritin concentrations at 12 GW were not reliable predictors of late pregnancy ID. However, sTfR, with a cutoff value of 1.018 μg/mL was the best iron marker measured in the first trimester to predict ID in late pregnancy, showing particular accuracy for women with overweight or obesity.

All BMI groups showed an overall decline in iron profile as pregnancy progressed, particularly during the first and second trimesters. Iron status deterioration persisted into the third trimester, particularly for women with obesity and high inflammation. These results were observed independently of the vitamin D dose received in the original intervention study. Our findings are in line with several studies that have shown a poorer iron status in pregnant women with obesity. Tussing-Humphrey et al. [38] showed lower ferritin concentrations in pregnant women with obesity in the late third trimester compared with women without obesity. Garcia-Valdes et al. [22] observed that although there were no differences in ferritin in early pregnancy, ferritin concentrations decreased and remained low by term in women with obesity, whereas concentrations recovered in normal-weight women. In addition, Flores-Quijano et al. [15] who longitudinally evaluated pregnant women who received 30 mg/d of elemental iron during pregnancy, showed a negative association between self-reported pre-pregnancy BMI and ferritin concentrations at 34 GW. Other research showed no differences in hemoglobin and ferritin concentrations between BMI groups [[15], [16], [17], [18], [19],^22^,^23^]. However, 2 of these studies did not evaluate markers in the third trimester of pregnancy [16,19]; 1 evaluated adolescent pregnancies only [23], and 2 did not report whether or not participants received iron supplementation [17,21]. Although Stoffel et al. [18] did not observe differences in ferritin concentrations between the BMI groups, the participants received a higher dose of iron supplementation (30–80 mg/d) compared with our study, but in addition and supporting our findings, also reported lower fractional iron absorption in women with obesity compared with normal weight. Iron status may seem similar across BMI groups in early pregnancy during routine assessments; however, our study shows that pregnant women with obesity experience a substantial deterioration of iron status as pregnancy progresses, placing them at heightened risk for IDA, increasing their susceptibility to severe obstetric and perinatal complications associated with this condition.

Hepcidin levels dropped significantly between the first and second trimesters in all BMI groups. In women with obesity, this downward trend continued into the third trimester, suggesting a sustained disruption in iron regulation throughout pregnancy. Although some studies have reported higher hepcidin levels in pregnant women with obesity [15,22,23,28], several others, including ours, have found no significant differences [[16], [17], [18], [19],^21^]. In our study, the persistently low hepcidin levels observed in the third trimester in women with obesity may reflect a more severe deterioration in iron status since early pregnancy. This is supported by lower serum iron and TSAT concentrations, as well as a more pronounced decline in hemoglobin. The deterioration continues into the third trimester, as evidenced by the persistent decline in hepcidin, along with a further depletion in ferritin and an increase in sTfR, all markers of worsening ID. It could be speculated that iron supplementation may have exacerbated the differences in iron status between women with and without obesity during pregnancy, as a result of a lower response to iron supplementation in women with obesity. Despite higher iron intake, impaired iron status has already been reported in women of reproductive age with central obesity (coincidentally a group that presents higher inflammation) compared with women without obesity. This suggests that the deeper deterioration of iron status in these women observed in the present analysis would have started before pregnancy [2]. Furthermore, in this analysis, compared with women without obesity, those with obesity had higher concentrations of proinflammatory markers in the first and second trimesters, but not in the third trimester. Only women with obesity with high inflammation at the beginning of pregnancy ended the pregnancy with lower ferritin concentrations. This could strongly suggest that the onset of this alteration in iron status occurred early in the first trimester or even before pregnancy. As pregnancy progresses, this iron status would worsen until reaching the third trimester, when coincidentally the inflammation is comparable to that of women without obesity, where iron depletion becomes significant enough to be evident with low ferritin, overriding any other inflammation-driven mechanism, including a possible increase in hepcidin.

In our analysis, sTfR concentrations were similar between BMI groups at all timepoints and between pregnant women with high and low inflammation scores; however, higher adiposity measures at 12 GW were associated with a more pronounced increase in sTfR throughout pregnancy, indicating a progression toward ID at 36 GW. Furthermore, in our analysis, sTfR at 12 GW was the strongest predictor of ID in the late third trimester in the entire sample, particularly in women with obesity. This finding is consistent with other studies reporting higher sTfR concentrations or positive associations with BMI in pregnancy [15,17,19,21,22,29]. Few studies have analyzed the accuracy of this marker for the prediction of ID in the third trimester. Åsberg et al. [32] showed that sTfR was a better predictor of ID defined as ferritin <15 μg/L in women with inflammation (CRP ≥ 5 mg/L). However, they did not present data on the gestational age at which these markers were evaluated (AUC = 0.788, 95% CI: 0.729, 0.856) [32]. Åkesson et al. [39] reported specificity of 100%, sensitivity of 71%, positive predictive value of 100%, and negative predictive value of 99% for sTfR in pregnant women at 11 and 36 GW for the prediction of ID defined as ferritin ≤12 μg/L and hemoglobin <102 g/L. Thus, these findings strongly suggest that higher adiposity is associated with a more pronounced decline in both iron stores and tissue iron in the third trimester, as evidenced by changes in sTfR. In particular, first-trimester sTfR was found to be the most effective predictor of ID in late pregnancy, especially in women with overweight/obesity, underlining the importance of early detection in this often underdiagnosed group.

Our findings indicate that by the end of the third trimester, 40% of pregnant women experienced ID (defined as ferritin <15 μg/L), with a notably higher prevalence among those with obesity (50%) compared with women of normal weight (30.2%), demonstrating that women with obesity are more likely to develop ID in late third trimester of pregnancy. These findings are in line with previous studies [15,16,22,29], including that of Mayasari et al. [16], who reported a significantly higher prevalence of ID defined as TSAT <16%, in women with obesity (70.8%) compared with those with overweight (53%) and normal weight (52.3%). Additionally, the prevalence of IDA, defined as TSAT <16% and hemoglobin <110 g/L, was higher in women with obesity compared with overweight (23.1 vs 11.7%, *P* = 0.002). Flores-Quijano et al. [15] also reported higher ID in obesity at <14 GW (defined by unadjusted ferritin <12 μg/mL: 3.8% in normal weight,7.5% in obesity; and defined by sTfR >2.11 mg/L, 1.9% in normal weight,7.5% in obesity). Flores-Quijano et al. [29] showed that the majority (78%) of women with ID defined as unadjusted ferritin <12 μg/L and sTfR> 4.74 mg/L had obesity. Garcia-Valdes et al. [22] reported an ID prevalence defined by sTfR>28.1 nmol/L of 2.6%, 3.2%, and 9.5% in normal weight, overweight, and obesity, respectively. Despite this, there are a few studies that have found similar prevalence of ID between pregnant women with and without obesity. Koenig et al. [17] showed no difference in ID prevalence between those of normal weight and obesity in the third trimester of pregnancy (defined with adjusted ferritin <12 μg/L; normal weight 17%, obesity 10%, *P* = 0.440). McCarthy et al. [40] analyzed 645 pregnant women without anemia recruited in the first trimester and followed up at 15, 20, and 33 GW, with similar prevalence rate and supplementation to the current analysis (ferritin <15 μg/L: 4.5%, 51.2%; ferritin <30 μg/L: 20.7%, 83.8% at 15 and 33 GW respectively); they found that obesity was not associated with ID, whether defined by ferritin (<12, <15, <30 μg/L) or by sTfR > 4.4 mg/L. However, ferritin was not adjusted for inflammation, which may mask ID in women with obesity and inflammation. Additionally, these 2 studies had low numbers of pregnant women with obesity, and iron status was assessed as late as 33 GW, in both cases. All the aforementioned studies have assessed adiposity exclusively through BMI, with many not adjusting ferritin concentrations for inflammation. The current study advances the field by not only examining iron status across BMI categories in early pregnancy and its trajectory throughout gestation, but also by providing robust evidence that high adiposity and centrally distributed fat, beyond BMI alone, are significantly associated with an increased risk of ID and impaired iron status, particularly in the late third trimester.

This study highlights the increased vulnerability of pregnant women with obesity to developing ID and anemia during pregnancy, especially in the third trimester. The mechanisms involved may be complex and begin even before pregnancy begins. There is growing evidence suggesting that non-pregnant women of reproductive age with central obesity have a higher prevalence of ID, defined by elevated sTfR concentrations, and especially IDA [2]. A proportion of these women may experience a chronic low-grade inflammatory state. In such cases, inflammatory markers like CRP, TNF-α, IL-6, and IL-1β can contribute to shortened RBC lifespan through processes such as antibody and complement deposition on erythrocytes, mechanical damage from fibrin in the microvasculature, and increased macrophage activity [41]. These cytokines also impair the bone marrow's response to erythropoietin, initially leading to reduced RBC production. As a compensatory mechanism, erythropoietin levels begin to rise and may stay elevated for some time, even while hemoglobin remains within normal limits [19,42]. Eventually, however, this response becomes insufficient, and hemoglobin levels begin to decline. When women with obesity and underlying inflammation become pregnant, they face a dramatic rise in iron requirements [43]. Yet, many are unable to meet these demands. In the first trimester, differences in iron status in women with obesity may not be apparent, as inflammation can mask deficiencies in routine clinical markers such as hemoglobin, RBC count, and ferritin [44]. As pregnancy advances, the growing iron demands expose the weaknesses in iron regulation and RBC production, particularly as the compensatory erythropoietin response nears exhaustion. In addition to inflammation, elevated hepcidin levels, previously observed in women of reproductive age with obesity [45,46], may further complicate iron regulation. These elevations, often driven by inflammatory cytokines like IL-6, IL-1, IL-22, and IFN-α [47], reduce iron availability by blocking its absorption and release. This may explain why, in our study, women with obesity had low serum iron and TSAT from the early stages of pregnancy. By the time these women reach the third trimester, the period with the highest iron requirements, they are already at a compromised physiological position. With impaired RBC production, reduced iron absorption [18], and a depleted iron status from the start, their ability to meet these critical demands may be compromised. This cascade not only affects maternal health but may also have lasting consequences for the developing fetus.

To acknowledge limitations, this analysis was observational in nature, which does not facilitate the determination of a causal relationship between obesity and the development of ID. Furthermore, a high dropout rate was observed after baseline; however, the dropouts did not differ in baseline hematological or iron status compared with those with complete data. Nonetheless, multiple imputation was applied to account for the missing data. In addition, this study was a secondary analysis based on different aims not directly related to the objectives of the primary study. Furthermore, we were unable to demonstrate differences in the iron markers in the umbilical cord samples, most likely due to lower sample size and insufficient statistical power. On the basis of non-central F-distribution of 0.068, we estimate that ≥1607 pregnant women would be needed in each BMI group to demonstrate differences in fetal ferritin concentrations.

One of the main strengths of this study is the wide range of iron and inflammatory markers analyzed at all timepoints, as well as their longitudinal analysis according to the different BMI groups. Additionally, the participants in this study only received the supplementation administered for the trial, giving the assurance that all received the same dose of oral iron. Another strength is the inflammation adjustment in ferritin concentrations applied, considering the strong influence of CRP on ferritin concentrations.

In conclusion, this study provides compelling evidence that obesity and elevated adiposity in early pregnancy significantly increase the risk of ID in the late third trimester, a relationship primarily mediated by chronic low-grade inflammation. Traditional biomarkers such as hemoglobin and ferritin in early pregnancy are poor predictors of later ID, whereas sTfR emerges as a robust early indicator of risk. Despite the substantial rise in iron requirements during pregnancy, current global dietary guidelines remain outdated, failing to recommend appropriate incremental adjustments for iron intake. Notably, in this cohort, 40% of women developed ID by late pregnancy, even while receiving 17 mg/d of elemental iron from 12 GW, regardless of BMI. This clearly indicates that 17 mg/d, the amount used in various pregnancy multivitamin supplements, is inadequate to meet the physiological demands of pregnancy, particularly in women with obesity. These findings underscore an urgent need to revise dietary recommendations to reflect the heightened iron requirements of pregnancy and to incorporate personalized guidance based on maternal risk factors. In parallel, public health policies must prioritize early detection, effective risk stratification, and proactive prevention of ID and IDA throughout gestation. Given the global burden of ID and the rising prevalence of overweight and obesity, addressing this dual challenge is critical to improving maternal and neonatal outcomes worldwide.

## Author contributions

The authors’ responsibilities were as follows – SPD, MSM, MAK, MTM: designed research; SPD: conducted research and analyzed data or performed statistical analysis; MTM: primary responsibility for final content; and all authors: wrote paper, read and approved the final manuscript.

## Data availability

Data described in the manuscript, code book, and analytic code will be made available on request pending.

## Funding

SPD is a PhD Researcher funded by the Department for the Economy scholarship, Ulster University. This research received funding from Solvotrin Therapeutics.

## Conflict of interest

MTM, MSM and MAK report financial support was provided by Solvotrin Therapeutics. MTM, MSM and MAK report a relationship with Solvotrin Therapeutics that includes: funding grants. ML reports a relationship with Solvotrin Therapeutics that includes: board membership, equity or stocks, and funding grants. ML has patent #WO2017158030A1 Compositions and methods for increasing iron intake in a mammal issued to Trinity College Dublin and Solvotrin Therapeutics. All other authors, declare that they have no known competing financial interests or personal relationships that could have appeared to influence the work reported in this paper.
